# Physical activity-related health and economic benefits of building walkable neighbourhoods: a modelled comparison between brownfield and greenfield developments

**DOI:** 10.1186/s12966-019-0775-8

**Published:** 2019-02-20

**Authors:** Belén Zapata-Diomedi, Claire Boulangé, Billie Giles-Corti, Kath Phelan, Simon Washington, J. Lennert Veerman, Lucy Dubrelle Gunn

**Affiliations:** 1 School of Medicine, Griffith University Gold Coast, Building 40, level 8, room 8.38, Gold Coast, QLD 4222 Australia; 2 RMIT University, Healthy Liveable Cities Group, Centre for Urban Research, Melbourne, Victoria Australia; 3 Infrastructure Victoria, Melbourne, Victoria Australia; 4 School of Civil Engineering, the University of Queensland, Brisbane, Queensland Australia; 5 Faculty of Medicine, School of Public Health, The University of Queensland, Brisbane, Queensland Australia; 6 Cancer Research Division, Cancer Council NSW, Woolloomooloo, New South Wales Australia

**Keywords:** Urban form, Built environment, Physical activity, Health and economic benefits, Health impact assessment, Evaluation, Public health, Brownfield, Greenfield, Development

## Abstract

**Background:**

A consensus is emerging in the literature that urban form can impact health by either facilitating or deterring physical activity (PA). However, there is a lack of evidence measuring population health and the economic benefits relating to alternative urban forms. We examined the issue of housing people within two distinct types of urban development forms: a medium-density brownfield development in an established area with existing amenities (e.g. daily living destinations, transit), and a low-density suburban greenfield development. We predicted the health and economic benefits of a brownfield development compared with a greenfield development through their influence on PA.

**Methods:**

We combined a new Walkability Planning Support System (Walkability PSS) with a quantitative health impact assessment model. We used the Walkability PSS to estimate the probability of residents’ transport walking, based on their exposure to urban form in the brownfield and greenfield developments. We developed the underlying algorithms of the Walkability PSS using multi-level multivariate logistic regression analysis based on self-reported data for transport walking from the Victorian Integrated Survey of Transport and Activity 2009–10 and objectively measured urban form in the developments. We derived the difference in transport walking minutes per week based on the probability of transport walking in each of the developments and the average transport walking time per week among those who reported any transport walking. We then used the well-established method of the proportional multi-cohort multi-state life table model to translate the difference in transport walking minutes per week into health and economic benefits.

**Results:**

If adult residents living in the greenfield neighbourhood were instead exposed to the urban development form observed in a brownfield neighbourhood, the incidence and mortality of physical inactivity-related chronic diseases would decrease. Over the life course of the exposed population (21,000), we estimated 1600 health-adjusted life years gained and economic benefits of A$94 million.

**Discussion:**

Our findings indicate that planning policies that create walkable neighbourhoods with access to shops, services and public transport will lead to substantial health and economic benefits associated with reduced incidence of physical inactivity related diseases and premature death.

**Electronic supplementary material:**

The online version of this article (10.1186/s12966-019-0775-8) contains supplementary material, which is available to authorized users.

## Introduction

Over one half of the world’s population (54%) now lives in cities [1], and trends suggest this proportion will reach 66% by 2050 [2]. Accommodating this rapid growth presents infrastructure and housing challenges, but also opportunities to create liveable places where people can be active and healthy. Creating cities that support healthy lifestyles is key to preventing chronic diseases [3–5]: the United Nations (UN) [6], UN Habitat [7] and the World Health Organisation (WHO) [8] have identified cities as central to creating a more sustainable future, and critical settings for promoting health and well-being [9].

Internationally, longitudinal studies and quasi-experiments have shown that residents who live in more walkable i.e., cycle- and pedestrian-friendly neighbourhoods have higher levels of physical activity (PA), principally through increased walking [10–12]. Indeed, recent European evidence also supports positive correlations for cycle network length and cycling levels [13]. Environments that promote active lifestyles have the potential to delay the onset of chronic diseases for which physical inactivity is now a well-established cause [14, 15].

Evidence on health and economic benefits of good urban form are needed to support interventions that improve population health by supporting PA (e.g. walking for transport). Few studies, however, have attempted to predict future health and economic consequences of urban form [16–18]. These studies are either quantitative health impact assessments or economic evaluations. Both methods quantify the net health benefits of changes in PA attributable to exposure to features of urban form (e.g. sidewalks, street connectivity and proximity to transit), however, only economic evaluations include intervention costs [16, 19]. Leaving costing aside, the literature has in common the following two-step process: (1) the effect estimate for the association of urban form exposure with health risk factors (health pathways), and (2) translation of changes in health risk factors into health and economic benefits.

The most commonly studied health pathways related to urban form are exposure to PA, traffic related air pollution and road trauma [16, 18]. Positive health outcomes relate to increased PA attributable to urban form interventions or scenarios that support walking or cycling, mostly for transport purposes [16–18]. If an intervention results in decreased numbers of private motor vehicle trips then exposure to air pollution can also be assessed by the positive impact to overall population health from improved air quality. However, those who take part in active travel (walking and cycling) are exposed to higher levels of air pollution compared with car drivers, which has a negative impact on their health. Such offsetting complexities arise in other ways too. High population density was found to be associated with both increased walking and poor air quality due to high concentration of pollutants in compact urban designs [20]. A shift from car driving to active transport is also associated with increased injuries and death (road trauma) [18], as pedestrians and cyclists are more vulnerable than car drivers to road trauma incidents [21, 22]. However, if safe infrastructure is provided (e.g. segregated cycle lanes) the risk to active travellers decreases [23, 24]. Nevertheless, positive health effects from improved levels of PA across populations appear to outweigh potential harms from exposure to air pollution and road trauma [18]. Although this may be different in highly polluted cities (e.g. Delhi) [25], it appears that the benefits of PA still outweigh potential harms in high income countries’ settings [25–27]. Hence, we focus on the association of urban form features with PA.

Despite the importance of urban form for population health, there is a dearth of reliable tools for assessing the health and economic impacts of urban developments. One such tool is the Health Economic Assessment Tool (HEAT) developed by the WHO [28, 29] that monetises health benefits from improved walking and cycling. HEAT translates changes in walking and cycling to health and economic outcomes as a result of reduced risk of all-cause mortality. However, transport and urban planners still need a tool to visualise and identify how different urban development patterns impact on PA and therefore on the burden from chronic disease and economic outcomes. For this study we combined a tool to aid the decision making process of urban and transport planners as to the PA impacts of alternative urban developments with a health prediction model to quantify health and economic benefits.

In Australia, in line with international trends, the Australian Bureau of Statistics (ABS) projected that city dwellers will increase from 15 million (66%) in 2013 to 27 million (72%) in 2053 [30]. Hence, we used our method to examine the issue of housing people within two distinct types of urban development: brownfield and greenfield. Brownfield development typically describes residential development of previously used land that is located within an existing urban area (e.g. industrial land) [31]. Greenfield development describes residential development of undeveloped land, generally farmland, located on the city fringes.

We compared:

1. Urban form features for the two different types of urban developments; and
2. PA related health- and economic benefits of housing a population in a brownfield compared with a greenfield development.

## Methods

### Context

Melbourne is the second largest city in Australia, with a population that is predicted to reach eight million by 2050 [32]. In 2017, the Victorian State government published ‘Plan Melbourne’ - a metropolitan strategic plan which addresses a wide range of challenges (transport congestion; employment, public transport and services accessibility; housing affordability; and environmental sustainability) [33]. The plan aims to influence housing supply within the existing urban area through industrial land redevelopment and new suburbs on the city’s fringe. The plan includes an aspirational target for 70% of new housing in established areas and 30% in new fringe suburbs.

### Study areas

**Fig. 1:**
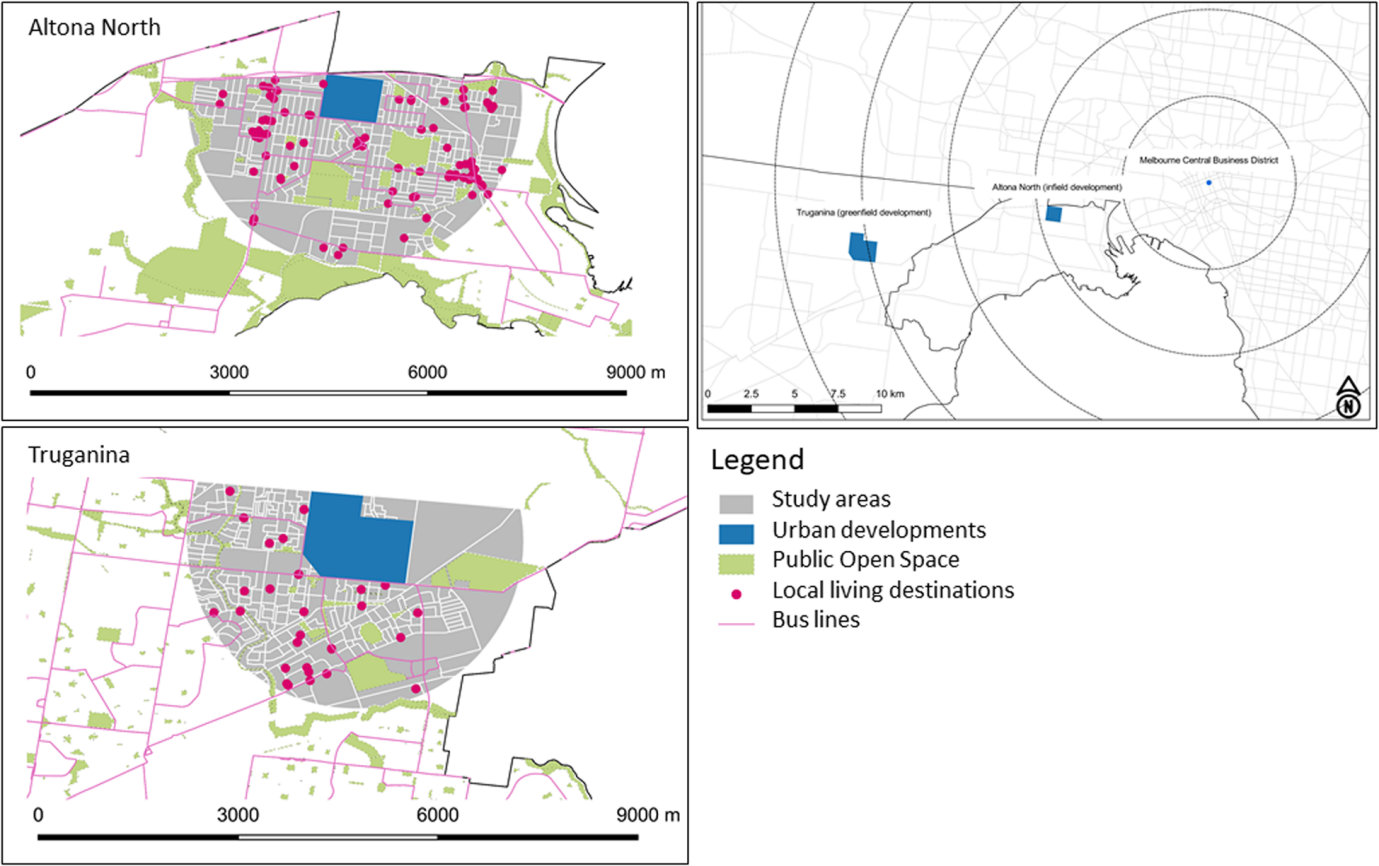
Map of the study areas showing their urban structure

Within Melbourne, our study areas include a planned brownfield redevelopment at Altona North and a new urban development in a suburb called Truganina (Fig. 1).

The planned brownfield redevelopment at Altona North is located within Melbourne’s established urban area approximately eight kilometres south west of the Melbourne Central Business District [34]. The development will provide 3000 dwellings housing approximately 7000 adults in a previously industrial area of 67 ha [35] (Table 2). In this analysis, a street network buffer zone of 3.2 km measured from the centre of the Altona North development was used as the ‘neighbourhood’ exposure area (Study areas in Fig. 1). In this area there are 21,600 adults without the development (Table 2). This size buffer captured the distance covered in 20 min walking and also the broader area with 12 categories of destinations (e.g. grocery stores and transit) [36]. The area to the north of the site was excluded from analysis due the presence of a freeway, which was a physical barrier to pedestrian movement.

We purposely selected a greenfield case study site with a similar number of houses and adult resident population. Truganina is located approximately 19 km (Euclidean distance) south west from the Melbourne Central Business District [37]. It is a suburban greenfield development on the city fringe. Similar to Altona North, we included only the residential area to the south of the site, as to the north, it was primarily farmland or partially developed land not suitable for residential purposes. We included a street network buffer zone of 3.2 km measured from the centre of the Truganina urban development site. Truganina has an adult population of approximately 21,000 people (Table 2).

We compared the urban form features and probability of transport walking for Altona North with and without the planned brownfield development to those in the greenfield suburban development of Truganina. We included Altona North without the development to show the uplift in amenity in the area from the new development. We simulated PA-related health and economic benefits of the Truganina adult population exposed to similar urban form features as those in the Altona North established area with the planned brownfield development. We present our results by urban form categories according to five of the six Ds from Ewing and Cervero [38]: density, design, diversity, destination accessibility and distance to transit (Table 1 and online Additional file 1: Table S1).

**Table 1:** Urban form features and their measurement

Urban form	Measurement^a^
Density	Number of dwellings per hectare (dw/ha). A second variable is a housing diversity score measured according to eight types (e.g. one storey terrace, two storey terrace, etc.), which serves as a proxy for compact living.
Design	Street connectivity was used as the measure of ‘design’ as it determines the ease of walking and proximity of destinations [38], measured as the number of intersections as an indicator of street connectivity [82].
Diversity	Diversity refers to the different uses of land in a given area. A land use mix score was developed based on the entropy measure of [83] where a high index represents a greater diversity of six land uses: commercial, education, industrial, parkland, residential and transport.
Destination accessibility	Destination accessibility was measured as a binary indicator representing the presence or absence of supermarkets, bus stops and train stations within 1 km, 400 m and 800 m respectively. These distances were chosen as they align to Victorian State government policy [84]. A second variable (local living destinations score) included 12 local living destinations (e.g. convenience store, supermarket, public transport stop, specialty food store, post office, bank, pharmacy, general practice/medical centre, dentist, community centre/hall, child care facility and library) to account for destination diversity which has been shown to influence walking behaviour [36].
Distance to transit	Measured as the presence of bus stops within 400 m and train stations within 800 m.

^a^Complete explanation in online Additional file 1: Table S1

### Simulation approach

We combined two previously developed models: the Walkability Planning Support System (Walkability PSS) [39] and a quantitative health impact assessment model [26]. The Walkability PSS estimates the probability that an adult participates in transport walking based on varying levels of exposure to a suite of urban form features. The quantitative health impact assessment model translates walking outcomes into health and economic benefits. The analysis compares a baseline scenario to an alternative scenario. The latter scenario is user-defined, and might be real developments, planned developments or hypothetical changes to the urban form.

### Walkability planning support System

The Walkability PSS is specially designed to explore and evaluate the walkability of an area as a result of urban form changes. The tool was developed in a series of workshops conducted in partnership with expert policy makers and urban planners [39]. The underlying algorithms were developed using multi-level multivariate logistic regression analysis based on data from the Victorian Integrated Survey of Transport and Activity 2009–10 (VISTA09). Earlier work examined the relationship between neighbourhood environments and transport-walking behaviours [40, 41]. More recent models include other transport mode outcomes such as cycling and public transport use that have been successfully used in several case studies [36]. These models are consistent with the literature and show that walkable environments discourage driving and support active transport behaviours.

In the interactive interface, users can work with a suite of spatial layers representing the underlying urban form features. Spatial layers include for example: road segments, land use types, public transport stops and frequency of services. Users can edit these layers and sketch onto the development plans via the digital map. As changes are made, the tool visualises the expected changes in transport walking. The development of the Walkability PSS and data sources are explained in detail elsewhere [39]. The model estimates the probability of undertaking any transport walking trips conditioned on urban form exposures (e.g. high access to transit versus low access).

For the quantitative health impact assessment model, we needed to assess the difference in the average number of minutes adults spent transport walking per week for the two case studies. We multiplied the probability of transport walking for each of the case studies with the average weekday minutes per person who participates in transport walking (Table 2). This product yielded the average time spent transport walking per week across the population for the studied areas (brownfield and greenfield), including both new residents and existing inhabitants. For the Altona North study area this included existing residents covered by the 3.2 km buffer (Fig. 1). The average difference between transport walking time per week between case studies was then used in the health impact assessment model as the potential increase in PA time per week of exposing a population in a greenfield development to the same urban form features as those observed in the brownfield development.

**Table 2:** Comparison of urban form features and probability of transport walking for each scenario

	Altona North	Altona North Developed	Truganina
Study area (ha)^a^	1645	1645	2800
Adult population^b^	21,618	27,065	20,970
Dwellings total	12,036	15,036 (12,036 + 3000)	9666
Urban form features [38]			
*Density*			
Gross dwelling per ha	7.3	9.1	3.5
Housing diversity (max = 8)^c^	6	6	5
*Design*			
Intersections per sq. km	39	54	33
*Distance to transit*			
Train station within 800 m	Yes	Yes	No
Bus stop within 400 m	Yes	Yes	Yes
*Destination accessibility*			
Supermarket within 1 km	Yes	Yes	No
Local living destinations score (max =12)^c^	11	11	8
*Diversity (land use)*			
Land use mix^c^	0.64	0.74	0.53
Probability of walking^d^			
	46%	48%	26%

*Ha* hectares, *Sq. km* squared kilometres

^a^Includes development and southern area within the 3.2 km street network buffer, as per Fig. 1

^b^Based on average number of adults per dwelling in Hobson Bay Local Government area [85] and Truganina State Suburb [85]

^c^Explained in the online Additional file 1: Table S1

^d^Estimated from the Walkability PSS model

### Transport behaviour data

We used data from the Victorian Integrated Survey of Travel and Activity 2009 (VISTA09) to estimate the average amount of time spent transport walking for those who reported any transport trips on the survey day [42]. VISTA includes data on a representative sample of the population and includes data on travel behaviour representing any weekday.

### Quantitative health impact assessment

The difference between minutes of weekly transport walking between the cases studies translates into differences in PA-related health and economic benefits through a proportional multi-cohort multi-state life table model (PMSLT) [26]. We simulated benefits over the remaining life course of the adult (≥ 18 years) population, with 2015 as the baseline year. We assumed that this population follows the same PA distribution at baseline as the overall Australian adult population [43]. The mechanisms of the PMSLT model are described in detail elsewhere [26] and are briefly explained in the online Additional file, which also details the input parameters (online Additional file 1: Table S3). Estimated outcomes are health-adjusted life years (HALYs), avoided new cases of chronic disease and mortality (incidence), healthcare cost savings, healthcare cost in added life years and total economic value. Literature with the same simulation approach [44] uses the term disability-adjusted life years (DALYs). We prefer the term HALYs to avoid confusion with the DALYs produced in burden of disease studies [45]. DALYs, as per the Global Burden of Disease studies are measured as years of life lost plus years lived with disability. Our HALYs are measured as life years adjusted with disability weights from the Global Burden of Disease Study that account for the loss of health-related quality of life. We estimated healthcare cost savings as the change in prevalence (ischemic heart disease, ischemic stroke and diabetes) or incidence (colon cancer and breast cancer) multiplied by the average healthcare cost of disease by age groups and sex [46]. We used the value of a statistical life year for Australia (A$182,000 in 2014 indexed to 2015 with the Consumer Price Index [47]) to monetise HALYs [48] and added these to net healthcare costs to produce a measure of total economic value. Applying the value of a statistical life year is widely used in the economic appraisal of transport interventions to monetise reduction in the risk of death [49].

### Uncertainty and sensitivity analysis

We used a 3% annual discount rate for healthcare costs and monetised HALYs [50] and tested the sensitivity of our results to discounting at 6% [51]. Ninety-five percent uncertainty intervals (UI) were determined for all outcome measures by Monte Carlo simulation (2000 iterations) using the Excel add-in tool Ersatz (Epigear, Version 1.34) (online Additional file 1: Table S3) [52]. Uncertainty parameters and their sources are presented in Additional file 1: Table S3.

## Results

### Urban form features of the different types of urban developments: Brownfield vs greenfield

Table 2 presents urban form features and the probability of transport walking for: Altona North (without the proposed urban development), Altona North Developed (once the proposed urban development is completed) and Truganina (greenfield). Overall, Altona North Developed had higher scores for all evaluated urban form features (described in greater detail below) and those commonly found to be associated with walking behaviour [53–55]. However, when compared with Altona North as is, the new development is not changing the destination mix and density significantly, as reflected in modest improvements in the probability of walking i.e., only 2 % (Table 2). Nevertheless, 3000 houses are built in an established area with existing amenities as opposed to building them in a greenfield area with low level of amenity. Specifically, the new residents benefit greatly from being located in an area with an existing variety of destinations (11 different types) as opposed to housing them in an area like Truganina which has a limited variety of destinations (8 different types). Hence, in what follows, we compared urban form features for Altona North Developed and Truganina to highlight the benefit of building urban developments within the city limits.

#### Density

Altona North Developed had a housing density of approximately nine dwellings per hectare, compared with three and a half in Truganina (Table 2). Although modest, this was achieved by a marginally higher housing diversity score (six in Altona North Developed compared with five in Truganina). As depicted in Table 2, with the proposed development, there will be 15,036 dwellings in Altona North in a total area of 1645 ha compared to 9666 dwellings in Truganina in a much larger area (2800 ha).

#### Design

The number of intersections was higher in Altona North Developed (54/sq.km) compared with Truganina (33/sq.km).

#### Diversity

Altona North Developed had a greater diversity of land uses with an index of 0.74 compared with Truganina’s 0.53.

#### Destination accessibility

Altona North Developed met the policy requirements of proximity to a train station, bus stop and supermarket and had a local living destination score of 11 (out of 12). Conversely, Truganina only had access to bus stops within the 400 m distance and eight of the local living destinations.

#### Transport walking

Across all VISTA09 adult survey participants, the mean daily transport walking time was 39 min and the probability of transport walking was 48% for Altona North Developed and 26% for Truganina. Hence, on average, an Altona North Developed resident was estimated to walk 131 min (48% * 39 min * 7 days) per week for transport purposes, while a resident in Truganina was estimated to walk 71 min per week (26% * 39 min * 7 days). Hence, the average difference in transport walking time among residents of the case study areas was 60 min per week.

### PA related health and economic benefits of alternative urban developments: Brownfield vs greenfield

Based on the changes in PA, we estimated that 1575 (95% Uncertainty Interval (UI), 1189 to 1962) HALYs could be gained if a population of 21,000 adults living in a greenfield development similar to Truganina are exposed to similar urban form features as a population living in an established neighbourhood like Altona North with the proposed urban development included. We estimated savings in healthcare costs attributable to preventing new cases of physical inactivity related chronic diseases of A$5.2 (95% UI, A$3 to A$7.5) million (2015). This was more than offset by increased healthcare costs of prolonged life of A$7.4 (95% UI, A$5.1 to A$9.7) million (2015). We also monetised HALYS using the value of a statistical life year, and calculated net healthcare cost to produce an overall metric of economic value. Our result of A$94 (95% UI, A$71 to A$117) million (2015) shows that healthcare cost changes are small when compared with the monetised value of HALYs.

**Fig. 2:**
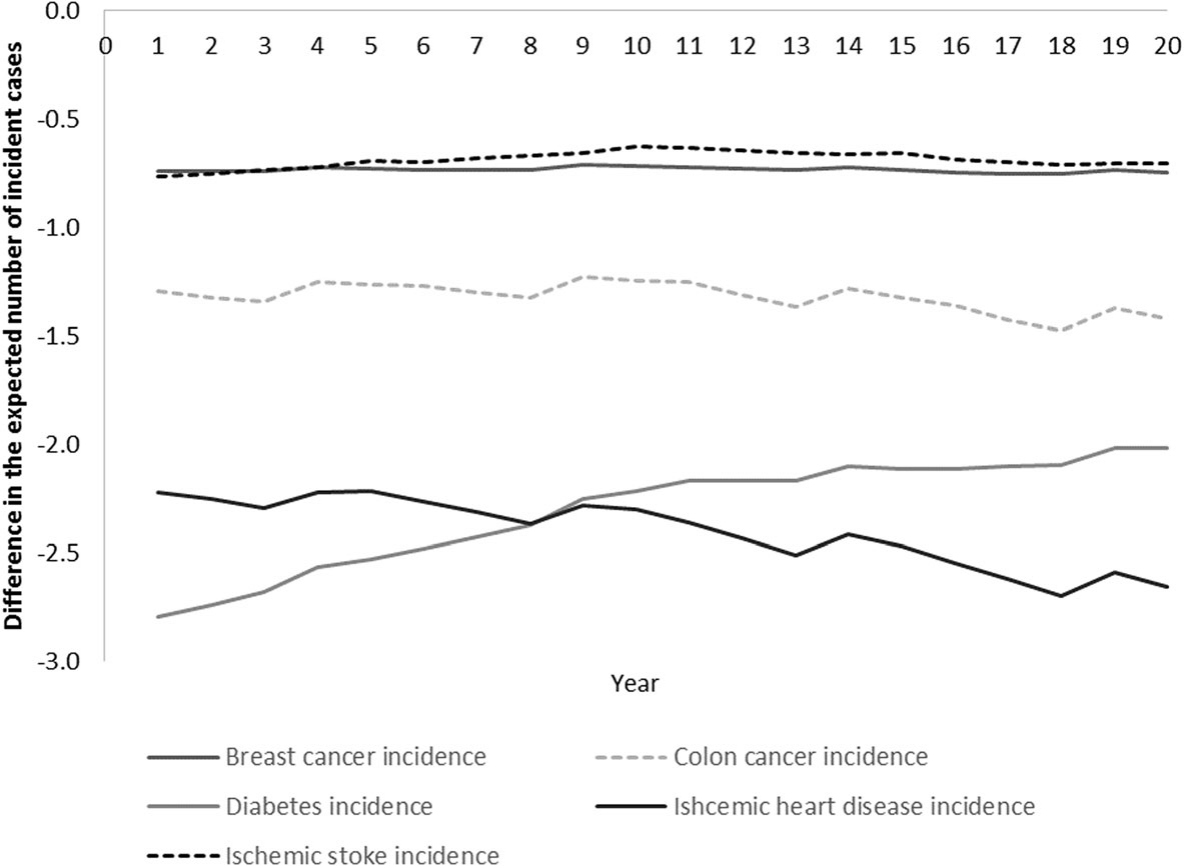
Difference in the expected number of incident cases per year over years 1 to 20 between Truganina and Altona North Developed as a result of different levels of PA (21,000 adults)

**Fig. 3:**
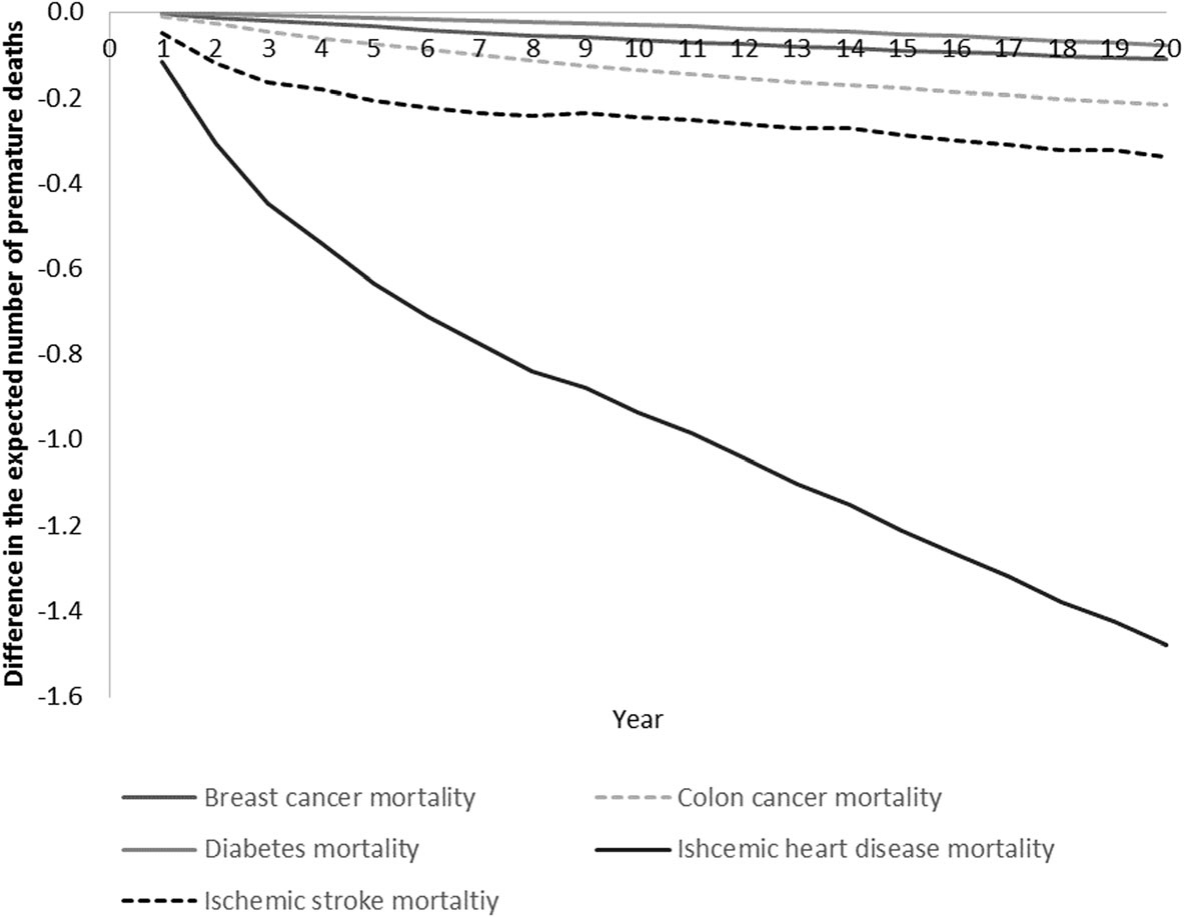
Difference in the expected number of premature deaths over years 1 to 20 between Truganina and Altona North Developed as a result of different levels of PA (21,000 adults)

We also simulated important decreases in incidence and mortality for breast cancer, colon cancer, diabetes, ischemic stroke and ischemic heart disease. Figures 2 and 3 depict the change in the burden of disease in numbers (y-axis) for the first 20 years (x-axis) of the model. The greatest health benefits are from a reduction in incidence attributable to ischemic heart disease and diabetes and mortality from ischemic heart disease and ischemic stroke.

Our results are sensitive to the choice of discounting, with higher discount rates resulting in lower present values (online Additional file 1: Table S4).

## Discussion

### Principal findings

We predicted the PA-related health and economic benefits of housing an adult population in a higher density brownfield development located in an established area with existing amenities compared with housing them in a greenfield outer suburban development. We brought together two separate models: the Walkability PSS model and a quantitative health impact assessment model. Our findings suggest important health benefits from a reduction in the burden of chronic disease attributable to physical inactivity, representing approximately one extra month of life in full health and an economic benefit of A$4500 per person over their life course.

Nevertheless, this may underestimate the potential health benefits of well-located brownfield developments. As was observed, the proposed new development brought only a small improvement to the established area (Table 2). Notably, there were only modest improvements in levels of density in Altona North from 7 dwellings per hectare for Altona North without the urban development to 9 dwellings per hectare with the development. This is despite the fact, that the density within the proposed development itself is 45 dwelling per hectare [56]. Our study highlights that while an new urban development may itself create a medium development [57], the very low densities observed show that density standards are not being implemented in practice. Moreover, the established area - Altona North - lags behind in the design features that promote walking compared with the most walkable areas in Melbourne. We identified high walkable neighbourhoods in Melbourne by filtering VISTA09 survey participants at the 75th percentile based on the frequency of transport walking trips and matched corresponding urban form features. We then re-ran our analysis in these areas and found that if our population of interest (21,000 adults living in Truganina) were instead exposed to urban form features observed in the most walkable areas in Melbourne, the overall health benefits would further improve by approximately 40% (online Additional file 1: Table S5 and S6). This highlights the importance of combining higher densities, with all the other Ds in our model: design, distance to transit, diversity and destination accessibility.

### Strengths and limitations

Strengths of this study include that, to the authors’ knowledge, this is the first to quantify health and economic benefits of housing a population in an established neighbourhood with a proposed brownfield urban development compared to housing them on the city fringes. We presented results for the probability of transport walking for the established neighbourhood (Altona North) with and without the brownfield development which provided a clear indication of the improvements to local amenity arising from a planned urban development. In our study, we combined changes in urban form simultaneously, as opposed to the summation of the effect of individual changes as presented in past literature. The few examples of composite or combined interventions [25, 58, 59] relied on the summation of individual changes made to the urban form on PA applying effect estimates from the literature [25, 59] or based on their own statistical models [58]. For example, Boarnet et al. [58] developed a statistical model and linked it to an economic evaluation framework to estimate the monetised PA-related health benefits of walkable places and found monetised health gains ranging from US$2 to US$24 million for a population of 5000 adults from a reduction in the risk of death. If we translate our results to 5000 people, we get approximately A$22 million, which is within the range found by Boarnet et al. We conducted our statistical analysis based directly on local travel data to derive our effect estimates adjusting for simultaneous changes to urban form. Past studies have taken two main approaches to predict health and economic benefits of urban form: static analysis, and life table analysis [17]. Most literature uses the static approach based on the comparative risk assessment (CRA) method [18] developed by the WHO [60]. CRA predicts the change in the burden of disease (e.g. disability-adjusted life-years, years of life lost) resulting from changes in health-related risk factors. In our study we used the proportional multi-state life table (MSLT) modelling approach to predict the potential effect on length and quality of life resulting from urban design interventions. Compared to the CRA approach, we were able to model multiple diseases simultaneously [61], avoiding the overestimation of results from summing health outcomes of each disease as done in the CRA method. In addition, CRA studies over estimate results when they calculate disability-adjusted life-years based on years of life lost (YLL) from Global Burden of Disease studies. This is because such YLLs are based on the highest attainable life expectancy observed in the world [62]. In our model, we used mortality specific to the State of Victoria, reflecting the local observed mortality. For example, Stevenson, Thompson [25] predicted health outcomes with the CRA method in six cities based on elasticity estimates from Ewing and Cervero [38] for the responsiveness of transport walking to changes in density, diversity of land uses and access to public transport. The authors found overall health gains of 420–826 disability-adjusted life-years per 100,000 population per year. If we translate our results to a population of 100,000 we get approximately 7500 HALYs over the life course, and approximately 200 per year, which is lower than Stevenson et al.

We used two separate models, and each presents limitation that should be highlighted. Our Walkability PSS model, like the rest of the Australian literature assessing urban design features and PA [54], relies on self-reported data for transport walking. Self-reported data is problematic due to recall bias and under/over reporting. We also estimated the probability of walking, and then translated this into duration using the average walking time across a travel survey. Our approach assumes an average behaviour which may be unrealistic. In addition, we did not control for self-selection – people selecting to live in neighbourhoods that facilitate walking. This may have led to overestimating the association between the urban form features and transport walking, but past literature showed the effect of controlling for self-selection to be small [55]. As to the quantitative health impact assessment model, limitations were previously described [26]. Here, we would like to highlight that our results are likely to be an underestimation as we only included five physical inactivity related diseases (ischemic heart disease, ischemic stroke, colon cancer, breast cancer and diabetes). Evidence from meta-analyses also relate PA to depression [63] and possibly dementia [64]. Furthermore, we did not differentiate health profiles of population groups, another source of underestimation. For example, in Australia, our most socio-economically disadvantaged group (defined by income) is over two times more likely to suffer from cardiovascular disease and have type 2 diabetes compared to the most advantaged group [65]. The most disadvantaged people in Australia also tend to be the most physically inactive [66]. Further, in our quantitative health model, we assumed that the effect of the intervention would translate into an increase in total PA. We did not account for potential replacement effects (e.g. shift from recreational-PA to transport-PA). Lastly, an overall limitation of our study is that we did not quantify the marginal costs of housing a population in a brownfield development in comparison with a greenfield development. The main reason for this was the lack of access to costing sources and the difficulty on establishing the boundaries of the study (what to include). A partial inclusion of costs, and benefits, may result in an unfair evaluation, as was the case with a study that we conducted assessing sidewalks [67]. Furthermore, our main aim was to demonstrate the potential physical activity related health and economic benefits of alternative housing options. An example of a recent complete economic evaluation is the study by Chapman et al. [68] in New Zealand where a benefit-cost ratio of approximately 10 was found for the interventions in active travel infrastructure. This study, in comparison to ours, assessed defined interventions, rather than whole developments, and included a wider range of benefits for health and the environment.

### Implications

Our study’s results demonstrate the importance of assessing the potential PA-related health and economic implications of alternative urban developments. It also highlights the potential of tools, like the Walkability PSS, for urban and transport planners to assess a priori the population health implication of their work when designing cities. As we showed here, providing housing for the expected growing population in brownfield developments in established areas with access to local amenities is considerably more favourable than the alternative of new low density outer suburban development in terms of population health and economic benefits. However, the success of brownfield sites will be optimised if they include mixed uses that add amenity to the local area, and if they are located in existing areas that already have sufficient densities to make those shops, services and transit viable. While our case studies were based in Australia, similar population growth challenges and urban development patterns plans are observed in other high-income countries such as New Zealand, United States and Canada. For instance, the Auckland Plan [69] aims for compact and high quality urban developments and provides guidelines for density levels in city centres and neighbourhoods for different types of urban areas (e.g. cities, metropolitan areas). For instance for cities, a gross density of 40–60 dwellings per hectare is recommended for neighbourhoods within the city limits and high densities in the city centre (50–200+ dwellings per hectare) [70].

Our results suggest that the health benefits of brownfield developments would be enhanced if they were located in areas with existing amenity and built to the levels set by the most walkable areas of Melbourne (e.g. in Melbourne Fitzroy, Carlton, Richmond). We also found that when developed the Altona North area including it’s 3.2 km catchment of existing residences achieved a gross density of nine dwellings per hectare (approximately 14 dwellings/hectare net [71]). This is just below current density targets of 15 dwellings/hectare net [72] and well below the 25 dwellings/hectare net required to support walking [36]. Without increased density across the new development and its catchment of existing residences, it is unlikely that local shops and services and public transport services would be economically viable. Thus, when designing brownfield developments in low density cities the balance between density and mixed land uses is an important consideration as sufficient density is necessary in surrounding neighbourhoods to support local shops, services and transit.

In the local context, our findings support the policy aspiration for 70% of Melbourne’s population growth to be accommodated in the established areas of the city, including in brownfield developments [33]. Notably, our analysis assessed the health benefits of relatively small areas (housing 21,000 adults in Melbourne). However, by 2050, it is estimated that Melbourne’s population may double from four to eight million. If our findings were applied to residents being housed in low density urban fringe developments across the city between now and 2050, the health and economic benefits would be significant. Future studies might also like to consider co-benefits associated with traffic congestion, greenhouse gas emissions and productivity.

In Australia, interest is growing in designing healthy and more sustainable walkable liveable communities [33, 73]. At the local government level, numerous examples exist of initiatives supporting health-enhancing behaviours, including PA [74–76] along with council plans to create healthy liveable communities [77, 78]. However, bringing about change across entire cities requires comprehensive integrated planning across multiple sectors [3], underpinned by more prescriptive evidence-based subdivision and residential development urban design guidelines or codes to create the walkable and pedestrian-friendly neighbourhoods. The Western Australia’s Liveable Neighbourhood guidelines provides an example of a State government effort to create pedestrian-friendly neighbourhoods that support healthy life styles [79]. An evaluation of the guidelines demonstrated that for every 10% increase in the implementation of the policy, residents’ likelihood of transport walking increased by 10% [80], however the policy was not fully implemented. The key focus therefore is good urban design policy that is well implemented.

## Conclusions

Well-located higher density brownfield developments in established areas with existing amenities are likely to produce better health outcomes and economic benefits compared with continuing to house people in low density developments on the urban fringe. The economic and health-related value of locating people in higher density brownfield developments can and should be estimated and be taken into account when legislating, guiding and designing urban forms to create healthy, liveable cities. Further efforts should be made to assess the impact of urban development patterns for population health a priori. Tools such as those in this study, could enhance urban and transport planning, and allow health departments to assess health impacts of city planning decision-making.

## Additional file

Additional file 1: Additional file. (DOCX 205 kb)

## Abbreviations

ABS: Australian Bureau of Statistics
CRA: Comparative Risk Assessment
DALYs: Disability-adjusted life years
HALYs: Health-adjusted life years
HEAT: Health economic assessment tool
PA: Physical activity
PMSLT: Proportional multi-cohort multi-state life table
UI: Uncertainty interval
UN: United Nations
VISTA09: Victorian Integrated Survey of Transport and Activity 2009–10
Walkability PSS: Walkability Planning Support System
WHO: World Health Organization
YLL: Years of life lost
