# Supplementary material for: Physical activity-related health and economic benefits of building walkable neighbourhoods: a modelled comparison between brownfield and greenfield developments
Source: Int J Behav Nutr Phys Act. 2019 Feb 20;16:11. doi: 10.1186/s12966-019-0775-8 (PMC6381620; doi:10.1186/s12966-019-0775-8)
Supplement: Supplementary file 1 — Additional file. (DOCX 205 kb) [file 12966_2019_775_MOESM1_ESM.docx]

**9Additional file**

**Physical activity-related health and economic benefits of building walkable neighbourhoods: A modelled comparison between brownfield and greenfield developments**

Belén Zapata-Diomedi, Claire Boulangé, Billie Giles-Corti, Kath Phelan, Simon Washington, J Lennert Veerman & Lucy Dubrelle Gunn

**Contents**

[1 Introduction 3](#_Toc532916410)

[2 Walkability PSS tool 4](#_Toc532916411)

[3 Quantitative health impact assessment 8](#_Toc532916412)

[4 Results of sensitivity scenarios 11](#_Toc532916413)

[5 Results of comparison with most walkable areas in Melbourne 12](#_Toc532916414)

[6 CHEERS checklist—Items to include when reporting economic evaluations of health interventions [36] 13](#_Toc532916415)

[7 References 16](#_Toc532916416)

**List of tables**

[Table 1 Spatial measures developed for this study, data sources and GIS techniques [2] 6](#_Toc532916417)

[Table 2 Adjusted regression coefficients for participation in transport-walking in the neighbourhood based on Boulange, Gunn [1] 8](#_Toc532916418)

[Table 3 Model input parameters 10](#_Toc532916419)

[Table 4 Results of sensitivity analyses (A$, 2015) 11](#_Toc532916420)

[Table 5 Comparison of urban form and probability of walking for Truganina compared to highly walkable areas in Melbourne. 12](#_Toc532916421)

[Table 6 Predicted walking-related health and economic outcomes highly walkable areas in Melbourne Metro versus Truganina (over the life course of 21,000 adults) (95 % uncertainty interval (UI)) 12](#_Toc532916422)

# Introduction

This document expands on the methods used in this study and on the results. In the first section, we further explain the methods and input parameters for the Walkability PSS model. In the second section, we briefly explain the quantitative health impact assessment model and its input parameters. In the third section, we present the results for our sensitivity analysis. In the fourth section, we present an additional analysis that compares exposing our population of interest to similar urban design as that observed in the most walkable areas in Melbourne. Lastly, we present the Consolidated Health Economic Evaluation Reporting Standards (CHEERS) checklist for our study.

# Walkability PSS tool

Planning [Support Systems](https://www-sciencedirect-com.ezproxy.lib.rmit.edu.au/topics/earth-and-planetary-sciences/support-systems) are spatially enabled computer based analytical tools designed to process [spatial data](https://www-sciencedirect-com.ezproxy.lib.rmit.edu.au/topics/earth-and-planetary-sciences/spatial-data). They are useful for modelling “what if” scenarios in support of planning analyses.

The Walkability Planning Support System (WPSS) used here was developed using ArcGIS 10.4 ([www.esri.com](http://www.esri.com/)) and its extension CommunityViz 5.1 ([www.city-explained.com](http://www.city-explained.com/)). The WPSS enables urban planners to visualise and explore urban form scenarios whilst calculating the potential impacts on the walkability of an area and the health of people living in those areas. In brief, the WPSS is designed for simulating changes in the urban form and measuring the impact these changes would have on transport walking behaviours.

As presented in Figure 1, data is loaded into a geodatabase, which is manipulated via CommunityViz when planners sketch scenarios of interest. The changes made in these scenarios are linked to a suite of custom formulae. The formulae extract information from the spatial data loaded in the geodatabase. These formulae calculate the urban form variables which include: land use mix; intersection density; dwelling density; housing diversity score; local living score; closest supermarket; closest train station; closest bus stop (see Table 1).


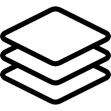

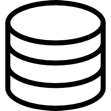


1. Spatial data is accessed from the geodatabase


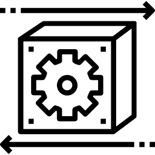

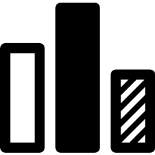


3. Custom formulas process information from the maps

4. Interactive charts presents performance indicators for each scenario


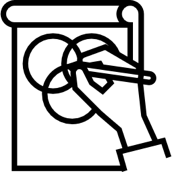


2. Planners sketch a series of planning scenario on the map display

Figure 1 - Workflow

After programming the urban form variables, the walkability indicator was developed using a logistic regression formula detailed below:

$$Estimate of P\left( y_{i}=1 \right|x_{i1, \ldots}x_{ip})= \frac{e^{(b_{0}+ b_{1} X_{i1}+\ldots{+ b}_{p} X_{ip})}}{1+ e^{(b_{0}+ b_{1} X_{i1}+\ldots{+ b}_{p} X_{ip})}}$$

In this formula, the effect of each urban form variable ($x_{k}$) is “weighted” by its regression coefficient ($b_{k}$). The formula measures the probability that an adult survey participant undertakes at least one or more transport walking trips (i.e. y=1) [1].

Coefficients from this model are presented in Table 2.

Table S1 Spatial measures developed for this study, data sources and GIS techniques [2]

| **Measure** | **Description** | **Data** |
| --- | --- | --- |
| Land Use Mix | The land use mix measure was calculated using the following entropy formula, which is adapted from Frank et al. [3] and is the same as the one used by Christian and colleagues [4]:  $LUM= -1(\sum_{i=1}^{n} pi*ln(pi))/ln(n)$  Five land use categories: residential, retail, commercial, industrial and “other” were used in the calculation. | A customised land use layer was created where parcel boundaries [5], zoning data [6], and geocoded business points data [7] were combined. The parcel boundaries data and zoning data were readily available nationally while the business points were available on a fee-for-service basis. |
| Connectivity | The connectivity measure was calculated using the following formula  $\frac{Count of 3-or more ways intersections}{Buffer area (in sq. km)}$ | Intersection data were derived from the VicMap Transport road centrelines [8]. |
| Dwelling density | The dwelling density measure was calculated using the following formula (gross dwelling density) and is the same as the one used by Badland and colleagues [9]. Gross density measure was selected because it is a measure of the place and appropriate when focussing on residential neighbourhoods.  $\frac{Sum of all dwellings}{Buffer area (in sq. km)}$ | Dwelling count data were sourced from the meshblock-level census data [10]. |
| Housing diversity | The housing diversity measure was calculated as the sum of the presence/absence of each of the following eight housing types in a buffer.  Standalone house  1 story terrace  2+ story terrace  0-2 story flat/unit/apartment  3 story flat/unit/apartment  4+ story flat/unit/apartment  Flat attached to a house  Other | Dwelling typology data were sourced from the meshblock-level census data [10]. |
| Local living destinations | The local living destination measure was calculated as the sum of the presence/absence of each of the following 12 destination categories in a buffer:   - Convenience destination (i.e. convenience store, newsagent, or petrol station) - Supermarket - Public transport stop (i.e. bus, tram, or train) - Speciality food destination (i.e. fruit and vegetable, meat, fish, or poultry store) - Post office - Bank - Pharmacy - General practice/medical centre - Dentist - Community centre or hall - Child care facility - Library | Destinations data were sourced from geocoded business points data [7]. |
| Closest supermarket | The closest supermarket measure was calculated as the smallest value in the matrix of network distances among home addresses and supermarkets points. | Supermarket data were sourced from geocoded business points data [7]. Street network data were derived from the VicMap Transport road centrelines [8]. |
| Closest train station | The closest train station measure was calculated as the smallest value in the matrix of network distances among home addresses and train station points. | Train station data were sourced from Public Transport Victoria data. Street network data were derived from the VicMap Transport road centrelines [8]. |
| Closest bus stop | The closest bus stop measure was calculated as the smallest value in the matrix of network distances among home addresses and bus stop points. | Bus stops data were sourced from Public Transport Victoria data. Street network data were derived from the VicMap Transport road centrelines [8]. |

Table S2 Adjusted [regression coefficients](https://www-sciencedirect-com.ezproxy.lib.rmit.edu.au/topics/earth-and-planetary-sciences/regression-coefficients) for participation in transport-walking in the neighbourhood based on Boulange, Gunn [1]

| **Urban form** | **Coefficient** | **(Std. Err.)** |
| --- | --- | --- |
| ***Street connectivity*** | 0.00350 | (1.52) |
| ***Land Use Mix*** | 0.409 | (1.67) |
| ***Local living destinations*** | 0.168*** | (8.22) |
| ***Housing diversity*** | 0.0945** | (2.64) |
| ***Dwelling density (gross)*** |  |  |
| *< 10.0 dph* | Ref. |  |
| *10.0 – 14.9 dph* | -0.341* | (-2.42) |
| *15.0 – 19.9 dph* | -0.0350 | (-0.21) |
| *20.0 – 29.9 dph* | 0.668** | (3.17) |
| *30.0 – 39.9 dph* | 1.022*** | (3.51) |
| *> 40 dph* | 0.927* | (2.46) |
| ***Distance to closest supermarket*** |  |  |
| *Over 1000 metres* | Ref. |  |
| *Between 500 metres and 1000 metres* | 0.0673 | (0.69) |
| *Within 500 metres* | 0.405*** | (3.63) |
| ***Distance to closest train station*** |  |  |
| *Over 800 metres* | Ref. |  |
| *Within 800 metres* | 0.224** | (2.70) |
| ***Distance to closest bus stop*** |  |  |
| *Over 400 metres* | Ref. |  |
| *Within 400 metres* | 0.296*** | (4.03) |
| ***Constant*** | -4.164*** | (-16.13) |

# Quantitative health impact assessment

The quantitative health impact assessment model is based on the proportional multi-state multi-cohort life table model originally developed for the Assessing Cost-Effectiveness in Prevention project (ACE Prevention) [11-14]. The model consists of a life table, a separate section for each of the modelled diseases and a section for population impact fraction (PIF) calculations. We included five diseases related to low levels of PA (ischemic heart disease, ischemic stroke, type 2 diabetes, colon cancer and breast cancer in women [15-17]). We modelled 5-year age groups by sex until everyone reaches the age of 100 or dies. The PIF ‘relative risk shift’ method was used to estimate the effect of changes in PA on disease incidence rates [18]. Changes in incidence impact on prevalent numbers of cases in later years, and consequently on years lived with disability and mortality. HALYs represent life years adjusted for disability attributable to disease and injury adjusted for the reduction in quality of life. Disability weights were derived from the Global Burden of Disease study (Table 2). The ‘relative risk shift’ method for calculating the PIF requires estimates of PA prevalence at baseline and for the intervention scenario, and corresponding relative risks (RR) of physical inactivity-related diseases. The Australian Bureau of Statistics collects data on four types of PA undertaken in the previous week by age and sex: walking for transport, walking for recreation, moderate PA (excluding walking) and vigorous PA [19]. From this we derived PA scores by multiplying mean time spent in each of the aforementioned PA categories by an assigned metabolic equivalent value (MET) [20], results were then summed. These scores were used to categorise people into those who were highly active (≥1,600 MET-minutes)/wk.), those who met the recommended level of activity (600 to <1,600 MET-minutes/wk.), those who were insufficiently active <600 MET-minutes/wk.), and those who were inactive (0 MET-minutes/wk.) [15]. We used Excel slope and intercept functions to fit RRs reported by Danaei, Ding [15] with RRs as the dependent variable and mean MET-minutes/wk. per PA category at baseline as the independent variable. We used the slope and intercept parameters to estimate RRs for the modelled scenarios with MET-minutes per PA category changing accordingly. RRs were estimated for a four-tier dose-response relationship (inactive, low active, moderately active and highly active) between PA and health outcomes. For modelling the relationship, categorical RRs were converted into continuous functions. Assuming non-linear associations between PA and health outcomes, we fitted log-linear functions with a power transformation of mean energy expenditure per PA category (inactive, low active, moderately active and highly active) serving as the independent variable and RRs reported in the source data serving as the dependent variable (0.5 for type 2 diabetes, ischemic heart diseases and breast cancer and 0.25 for ischemic stroke and colon cancer). Those in the highly active group had a RR of 1.00 in the source study, implying no additional benefit from extra PA. Because type 2 diabetes is a risk factor for cardiovascular disease, estimated RRs incorporated the increased risk of ischemic heart disease and ischemic stroke among those with type 2 diabetes. To avoid double counting we reduced the PIFs for PA with ischemic heart disease (14%) and ischemic stroke (8%) [21p. 711 Supplementary Material].

Healthcare costs are from the ACE Prevention project, which used data from a 2001 health expenditure study [22]. We indexed these to 2015 using the Health Price Index [23]. Healthcare costs and monetised HALYs were discounted at an annual rate of 3% [24]. Ninety-five percent uncertainty intervals were determined for all outcome measures by Monte Carlo simulation (2,000 iterations), using the Excel add-in tool Ersatz (Epigear, Version 1.34) [25]. The model was set up in Excel and a range of input data was required, as presented in Table 3, alongside inputs distributions to derive outcomes with uncertainty.

Table S3 Model input parameters

| **Input parameter** | **Uncertainty/Parameters** | **Source** |
| --- | --- | --- |
| **Quantitative health impact assessment model** |  |  |
| 2015 mortality rates and population numbers (Victoria)^1^ | N/A | Australia Bureau of Statistics [26], Australian Bureau of Statistics [27] |
| 2015 epidemiological data (prevalence, incidence, case fatality, prevalence, mortality and years lived with disability) (Australian)^2^ | N/A | Institute for Health Metrics and Evaluation (IHME) [28] |
| Disability weights | N/A | Derived from prevalence and years lived with disability from GBD 2015 [28] |
| Disease trend | N/A | Cancers: Australian Institute of Health and Welfare [29]. Ischemic heart disease, ischemic stroke and type 2 diabetes: Australian Institute of Health and Welfare [30]. Methods as per ACE Prevention [31]. |
| MET-minutes PA categories | Lognormal | National Nutrition and Physical Activity Survey Basic Confidentialised Unit Record File (CURF) [19] |
| Relative risk, PA | Normal (Ln RR)^3^ | Danaei et al. [15] |
| Relative risks of ischaemic heart disease and ischaemic stroke due to diabetes | Normal (Ln RR)^3^ | Asia Pacific Cohort Studies Collaboration [32] |
| Mediating effect factors for diabetes in the association physical activity-ischemic heart disease/ischemic stroke | Normal | GBD^d^ 2013 study [21p. 711 Supplementary Material] |
| MET-minutes (walking=3.5, cycling=6.8, moderate PA=5, vigorous PA=7.5) | N/A | Ainsworth et al. [20] for walking and cycling; Australian Bureau of Statistics [19] for walking, moderate and vigorous PA |
| Health care costs | N/A | ACE Prevention study [33] |
| Discount rate | N/A | Murray et al. [34] for health; Gold et al. [24] for health care costs |

**^1^** Population and mortality figures are for the State of Victoria, of which Melbourne is the capital city. Victoria was the smallest geographical division for which population and mortality data at the level of disaggregation required for this study was available.

^2^*Epidemiological data* for the five physical activity related diseases (ischemic heart disease, stroke, type 2 diabetes, colon cancer and breast cancer in women) were derived with the help of DISMOD II (available free of charge at http://www.epigear.com/index_files/dismod_ii.html) to obtain data in metrics not explicitly reported (incidence and case fatality from prevalence and mortality).

^3^ A modified version of the log of the relative risk function was used to avoid a skewed lognormal distribution [35]

# Results of sensitivity scenarios

Table S4 Results of sensitivity analyses (A$, 2015)

|  | **HALYS** | **Healthcare cost savings** | **Healthcare costs in added life years** | **Total monetised value** |
| --- | --- | --- | --- | --- |
| Baseline (healthcare and monetised HALYs at 3%) | 1,575 | -$5,193,049 | $7,416,659 | $93,888,405 |
| LUI | 1,189 | -$7,517,209 | $5,146,952 | $71,496,495 |
| UUI | 1,962 | -$3,040,276 | $9,752,107 | $116,939,981 |
| Discount costs (healthcare and monetised HALYs at 6%) | 1,575 | -$2,644,718 | $2,846,862 | $39,986,099 |
| LUI | 1,189 | -$3,753,728 | $1,902,096 | $30,376,283 |
| UUI | 1,962 | -$1,626,933 | $3,792,977 | $49,803,109 |

**LUI**: Lower uncertainty interval **UUI**: Upper uncertainty interval

# Results of comparison with most walkable areas in Melbourne

Table S5 Comparison of urban form and probability of walking for Truganina compared to highly walkable areas in Melbourne.

|  |  | **High walkable areas in Melbourne** | **Truganina** |
| --- | --- | --- | --- |
| **Urban form** |  |  |  |
| ***Density*** |  |  |  |
|  | Gross dwelling per ha | 29 | 3.5 |
|  | Housing diversity (max=8) | 8 | 5 |
| ***Design*** |  |  |  |
|  | Intersections per sq. km | 170 | 33 |
| ***Distance to transit*** |  |  |  |
|  | Train station within 800m | Yes | No |
|  | Bus stop within 400m | Yes | Yes |
| ***Destination accessibility*** |  |  |  |
|  | Supermarket within 1km | Yes | No |
|  | Local living destinations score (max =12) | 12 | 8 |
| ***Diversity*** |  |  |  |
|  | Land use mix | 0.81 | 0.53 |
| **Probability of walking** |  |  |  |
|  |  | 68% | 26% |

Table S6 Predicted walking-related health and economic outcomes highly walkable areas in Melbourne Metro versus Truganina (over the life course of 21,000 adults) (95 % uncertainty interval (UI))

|  | **HALYS** | **Healthcare cost savings** | **Healthcare costs in added life years** | **Total monetised value** |
| --- | --- | --- | --- | --- |
| Baseline (healthcare costs and monetised HALYs at 3%) | 2,229 | -$6,842,086 | $10,398,854 | $132,523,883 |
| LUI | 1,760 | -$9,428,635.15 | $7,541,714 | $105,746,349 |
| UUI | 2,714 | -$4,156,294.62 | $13,383,085 | $161,192,013 |
| Discount costs (healthcare costs and monetised HALYs at 6%) | 2,229 | -$3,493,558 | $3,975,656 | $56,374,883 |
| LUI | 1,760 | -$4,711,504 | $2,798,667 | $44,995,151 |
| UUI | 2,714 | -$2,224,920 | $5,196,738 | $68,461,183 |

**LUI**: Lower uncertainty interval **UUI**: Upper uncertainty interval

# CHEERS checklist—Items to include when reporting economic evaluations of health interventions [36]

**Note: we used the CHEERS checklist as this is the most suitable available checklist. However, our study is a modelling study of the potential health and economic implications of urban form and not a complete economic evaluation.**

| **Section/item** | **Item No** | **Recommendation** | **Reported on page No/ line No** |
| --- | --- | --- | --- |
| **Title and abstract** | | | |
| Title | 1 | Identify the study as an economic evaluation or use more specific terms such as “cost-effectiveness analysis”, and describe the interventions compared. | Title in Page 1, line 1  *Comment: we identified the outcomes (health and economic) and study type (modelling study) in the title.* |
| Abstract | 2 | Provide a structured summary of objectives, perspective, setting, methods (including study design and inputs), results (including base case and uncertainty analyses), and conclusions. | Page 2 and 3 |
| **Introduction** | | | |
| Background and objectives | 3 | Provide an explicit statement of the broader context for the study. | Page 4 and 5 |
|  |  | Present the study question and its relevance for health policy or practice decisions. | Page 5 and 6  *Comment: we framed our work as relevant for urban and transport planners. We do not present research questions but objectives.* |
| **Methods** | | | |
| Target population and subgroups | 4 | Describe characteristics of the base case population and subgroups analysed, including why they were chosen. | Page 6 and 7:  Subheading: Study areas |
| Setting and location | 5 | State relevant aspects of the system(s) in which the decision(s) need(s) to be made. | N/A |
| Study perspective | 6 | Describe the perspective of the study and relate this to the costs being evaluated. | N/A  *Comment: this is not a complete economic evaluation, more like a quantitative health impact assessment* |
| Comparators | 7 | Describe the interventions or strategies being compared and state why they were chosen. | Page 6 and 7:  Subheading: context and Study areas |
| Time horizon | 8 | State the time horizon(s) over which costs and consequences are being evaluated and say why appropriate. | Page 10: Subheading Quantitative health impact assessment |
| Discount rate | 9 | Report the choice of discount rate(s) used for costs and outcomes and say why appropriate. | Page 11: Subheading Uncertainty and sensitivity analysis |
| Choice of health outcomes | 10 | Describe what outcomes were used as the measure(s) of benefit in the evaluation and their relevance for the type of analysis performed. | Page 10 and 11: Subheading Quantitative health impact assessment |
| Measurement of effectiveness | 11a | *Single study-based estimates:*Describe fully the design features of the single effectiveness study and why the single study was a sufficient source of clinical effectiveness data. | N/A |
|  | 11b | *Synthesis-based estimates*: Describe fully the methods used for identification of included studies and synthesis of clinical effectiveness data. | N/A |
| Measurement and valuation of preference based outcomes | 12 | If applicable, describe the population and methods used to elicit preferences for outcomes. | N/A |
| Estimating resources and costs | 13a | *Single study-based economic evaluation:* Describe approaches used to estimate resource use associated with the alternative interventions. Describe primary or secondary research methods for valuing each resource item in terms of its unit cost. Describe any adjustments made to approximate to opportunity costs. | N/A |
|  | 13b | *Model-based economic evaluation:*Describe approaches and data sources used to estimate resource use associated with model health states. Describe primary or secondary research methods for valuing each resource item in terms of its unit cost. Describe any adjustments made to approximate to opportunity costs. | Additional file  Table 3 |
| Currency, price date, and conversion | 14 | Report the dates of the estimated resource quantities and unit costs. Describe methods for adjusting estimated unit costs to the year of reported costs if necessary. Describe methods for converting costs into a common currency base and the exchange rate. | Page 10: Subheading Quantitative health impact assessment  And Page 11: Subheading Uncertainty and sensitivity analysis |
| Choice of model | 15 | Describe and give reasons for the specific type of decision-analytical model used. Providing a figure to show model structure is strongly recommended. | N/A  Reported in previous studies using the same modelling approach |
| Assumptions | 16 | Describe all structural or other assumptions underpinning the decision-analytical model. | Pages 9, 10 and 11:  Subheading Simulation Approach |
| Analytical methods | 17 | Describe all analytical methods supporting the evaluation. This could include methods for dealing with skewed, missing, or censored data; extrapolation methods; methods for pooling data; approaches to validate or make adjustments (such as half cycle corrections) to a model; and methods for handling population heterogeneity and uncertainty. | N/A  Modelling methods have been extensively described in past research [14]. |
| **Results** | | | |
| Study parameters | 18 | Report the values, ranges, references, and, if used, probability distributions for all parameters. Report reasons or sources for distributions used to represent uncertainty where appropriate. Providing a table to show the input values is strongly recommended. | Pages 14 and 15.  Additional file Tables 2 and 4. |
| Incremental costs and outcomes | 19 | For each intervention, report mean values for the main categories of estimated costs and outcomes of interest, as well as mean differences between the comparator groups. If applicable, report incremental cost-effectiveness ratios. | Additional file Tables 5 and 6. |
| Characterising uncertainty | 20a | *Single study-based economic evaluation:*Describe the effects of sampling uncertainty for the estimated incremental cost and incremental effectiveness parameters, together with the impact of methodological assumptions (such as discount rate, study perspective). | N/A |
|  | 20b | *Model-based economic evaluation:*Describe the effects on the results of uncertainty for all input parameters, and uncertainty related to the structure of the model and assumptions. | Uncertainties are related to inputs and results are reported including uncertainty intervals. |
| Characterising heterogeneity | 21 | If applicable, report differences in costs, outcomes, or cost-effectiveness that can be explained by variations between subgroups of patients with different baseline characteristics or other observed variability in effects that are not reducible by more information. | N/A |
| **Discussion** | | | |
| Study findings, limitations, generalisability, and current knowledge | 22 | Summarise key study findings and describe how they support the conclusions reached. Discuss limitations and the generalisability of the findings and how the findings fit with current knowledge. | Page 15-21. |
| **Other** | | | |
| Source of funding | 23 | Describe how the study was funded and the role of the funder in the identification, design, conduct, and reporting of the analysis. Describe other non-monetary sources of support. | Page 22: Subheading Funding |
| Conflicts of interest | 24 | Describe any potential for conflict of interest of study contributors in accordance with journal policy. In the absence of a journal policy, we recommend authors comply with International Committee of Medical Journal Editors recommendations. | Page 22: Subheading Competing interest. |

# References

1. Boulange C, Gunn L, Billie G-C, Mavoa S, Pettit C, Badland H. Examining associations between urban design attributes and transport mode choice for walking, cycling, public transport and private motor vehicle trips. J Transp Health. 2017;6:155-66.

2. Boulange C. The Walkability Planning Support System. An evidence-based tool to design healthy communities: The University of Melbourne; 2016.

3. Frank LD, Schmid TL, Sallis JF, Chapman J, Saelens BE. Linking objectively measured physical activity with objectively measured urban form: Findings from SMARTRAQ. American Journal of Preventive Medicine. 2005;28(2, Supplement 2):117-25.

4. Christian H, Bull F, Middleton N, Knuiman M, Divitini M, Hooper P, et al. How important is the land use mix measure in understanding walking behaviour? Results from the RESIDE study. International Journal of Behavioral Nutrition and Physical Activity. 2011;8(55):doi:10.1186/479-5868-8-55.

5. Department of Sustainability and Environment, State Government of Victoria. VicMap Property. 2012.

6. Department of Sustainability and Environment, State Government of Victoria. VicMap Planning. 2012.

7. Pitney Bowes Ltd. Axiom business points. 2014.

8. Department of Sustainability and Environment, State Government of Victoria. VicMap Transport. 2012.

9. Badland H, Mavoa S, Boulangé C, Eagleson S, Gunn L, Stewart J, et al. Identifying, creating, and testing urban planning measures for transport walking: Findings from the Australian national liveability study. Journal of Transport & Health. 2016.

10. Australian Bureau of Statistics. Census of Population and Housing: Mesh Block Counts, 2011. <http://www.abs.gov.au/ausstats/abs@.nsf/Lookup/2074.0main+features12011>. 2011.

11. Barendregt JJ, Van Oortmarssen GJ, Van Hout BA, Van Den Bosch JM. Coping with multiple morbidity in a life table. Math Popul Stud. 1998;7(1):29-49.

12. Barendregt JJ, Oortmarssen vGJ, Murray CJ, Vos T. A generic model for the assessment of disease epidemiology: the computational basis of DisMod II. Popul Health Metr. 2003;1(1):4-.

13. Cobiac LJ, Vos T, Barendregt JJ. Cost-effectiveness of interventions to promote physical activity: a modelling study. Plos Med. 2009;6(7):e1000110-e.

14. Vos T, Carter R, Barendregt JJ, C. M, Veerman J, Magnus A, et al. Assessing Cost-Effectiveness in Prevention (ACE-Prevention): Final Report. University of Queensland, Brisbane and Deakin University, Melbourne; 2010.

15. Danaei G, Ding EL, Mozaffarian D, Taylor B, Rehm J, Murray CJL, et al. The preventable causes of death in the United States: comparative risk assessment of dietary, lifestyle, and metabolic risk factors. PLoS Med. 2009;6(4):e1000058.

16. Bull FC, Armstrong TP, Dixon T, Ham S, Neiman A, Pratt M. Physical inactivity. Comparative quantification of health risks: Global and regional burden of disease due to selected major risk factors Volume 1. 2004:729 - 881.

17. Kyu HH, Bachman VF, Alexander LT, Mumford JE, Afshin A, Estep K, et al. Physical activity and risk of breast cancer, colon cancer, diabetes, ischemic heart disease, and ischemic stroke events: systematic review and dose-response meta-analysis for the Global Burden of Disease Study 2013. BMJ. 2016;354.

18. Barendregt JJ, Veerman JL. Categorical versus continuous risk factors and the calculation of potential impact fractions. J Epidemiol Community Health. 2010;64(3):209-12.

19. Australian Health Survey: Physical Activity, 2011-12 [Internet]. 2015 [cited 22 September 2015]. Available from: <http://www.abs.gov.au/ausstats/abs@.nsf/Lookup/D4495467B7F7EB01CA257BAC0015F593?opendocument>.

20. Ainsworth BE, Haskell WL, Herrmann SD, Meckes N, Bassett DR, Jr., Tudor-Locke C, et al. 2011 compendium of physical activities: a second update of codes and MET values. Med Sci Sports Exerc. 2011;43(8):1575-81.

21. GBD 2013 Risk Factors Collaborators. Global, regional, and national comparative risk assessment of 79 behavioural, environmental and occupational, and metabolic risks or clusters of risks in 188 countries, 1990-2013: A systematic analysis for the Global Burden of Disease Study 2013. The Lancet. 2015;386(10010):2287-323.

22. Australian Institute of Health and Welfare. Disease costs and impact study data. Australian Institute of Health and Welfare; 2001.

23. Australian Institute of Health and Welfare. Health expenditure Australia 2014–15. Canberra: AIHW; 2016.

24. Gold MR. Cost-effectiveness in health and medicine. New York: Oxford University Press; 1996.

25. Barendregt JJ. EpiGear International. <http://www.epigear.com/index_files/prevent.html>. 2012. Accessed 1 March 2015.

26. Quarterly Population Estimates (ERP), by State/Territory, Sex and Age [Internet]. Australian Bureau of Statistics. 2017. Available from: <http://stat.data.abs.gov.au/Index.aspx?DataSetCode=ERP_QUARTERLY>.

27. Deaths, Australia, 2015 [Internet]. Australian Bureau of Statistics. 2017 [cited 13 October 2017]. Available from: <http://www.abs.gov.au/AUSSTATS/abs@.nsf/DetailsPage/3302.02015?OpenDocument>.

28. Global Burden of Disease Study 2015 [Internet]. 2016 [cited 15 June 2017]. Available from: <http://ghdx.healthdata.org/gbd-results-tool>.

29. Cancer mortality trends and projections: 2014 to 2025 [Internet]. 2013 [cited 13 October 2017]. Available from: <https://www.aihw.gov.au/reports/cancer/cancer-mortality-trends-and-projections-2014-to-2025/data>.

30. General Record of Incidence of Mortality (GRIM) books [Internet]. Australian Institute of Health and Welfare, . 2017 [cited 13 October 2017]. Available from: <https://www.aihw.gov.au/reports/life-expectancy-death/grim-books/contents/grim-books>.

31. Vos T, Carter R, Barendregt J, Mihalopoulos C, Veerman J, Magnus A, et al. Assessing Cost-Effectiveness in Prevention (ACE-Prevention): Final Report. Univresity of Queensland; 2010.

32. Asia Pacific Cohort Studies Collaboration. The Effects of Diabetes on the Risks of Major Cardiovascular Diseases and Death in the Asia-Pacific Region. Diabetes Care. 2003;26(2):360-6.

33. Vos T, Carter R, Barendregt JJ, C. M, Veerman J, Magnus A, et al. Assessing Cost-Effectiveness in the Prevention of Non-Communicable Disease (ACE–Prevention) Project 2005–09-Economic Evaluation Protocol. Brisbane, Australia: University of Queensland; 2007.

34. Murray CJL, Ezzati M, Flaxman AD, Lim S, Lozano R, Michaud C, et al. GBD 2010: design, definitions, and metrics. The Lancet. 2012;380(9859):2063-6.

35. Barendregt JJ. The effect size in uncertainty analysis. Value in health : the journal of the International Society for Pharmacoeconomics and Outcomes Research. 2010;13(4):388-91.

36. Husereau D, Drummond M, Petrou S, Carswell C, Moher D, Greenberg D, et al. Consolidated Health Economic Evaluation Reporting Standards (CHEERS)--explanation and elaboration: a report of the ISPOR Health Economic Evaluation Publication Guidelines Good Reporting Practices Task Force. Value in health : the journal of the International Society for Pharmacoeconomics and Outcomes Research. 2013;16(2):231-50.
